# Heterofucans from the Brown Seaweed *Canistrocarpus cervicornis* with Anticoagulant and Antioxidant Activities

**DOI:** 10.3390/md9010124

**Published:** 2011-01-24

**Authors:** Rafael Barros Gomes Camara, Leandro Silva Costa, Gabriel Pereira Fidelis, Leonardo Thiago Duarte Barreto Nobre, Nednaldo Dantas-Santos, Sara Lima Cordeiro, Mariana Santana Santos Pereira Costa, Luciana Guimaraes Alves, Hugo Alexandre Oliveira Rocha

**Affiliations:** 1 Laboratory of Biotechnology of Natural Polymers (BIOPOL), Department of Biochemistry, Federal University of Rio Grande do Norte (UFRN), Natal-RN, Brazil; E-Mails: rafael_bgc@yahoo.com.br (R.B.G.C.); gabrielfideliss@gmail.com (G.P.F.); leo_dnobre@yahoo.com.br (L.T.D.B.N.); nednaldod@hotmail.com (N.D.-S.); sara-cordeiro@hotmail.com (S.L.C.); marispc_bio@yahoo.com.br (M.S.S.P.C.); lucianagalves@hotmail.com (L.G.A.); 2 Federal Institute of Education, Science and Technology of Rio Grande do Norte (IFRN), Santa Cruz-RN, Brazil; E-Mail: leandro-silva-costa@hotmail.com

**Keywords:** *Dictyota cervicornis*, fucoidan, free radicals, sulfated polysaccharides

## Abstract

Fucan is a term used to denominate a family of sulfated polysaccharides rich in sulfated l-fucose. We extracted six fucans from *Canistrocarpus cervicornis* by proteolytic digestion followed by sequential acetone precipitation. These heterofucans are composed mainly of fucose, glucuronic acid, galactose and sulfate. No polysaccharide was capable of prolonging prothrombin time (PT) at the concentration assayed. However, all polysaccharides prolonged activated partial thromboplastin time (aPTT). Four sulfated polysaccharides (CC-0.3/CC-0.5/CC-0.7/CC-1.0) doubled aPTT with only 0.1 mg/mL of plasma, only 1.25-fold less than Clexane^®^, a commercial low molecular weight heparin. Heterofucans exhibited total antioxidant capacity, low hydroxyl radical scavenging activity, good superoxide radical scavenging efficiency (except CC-1.0), and excellent ferrous chelating ability (except CC-0.3). These results clearly indicate the beneficial effect of *C. cervicornis* polysaccharides as anticoagulants and antioxidants. Further purification steps and additional studies on structural features as well as *in vivo* experiments are needed to test the viability of their use as therapeutic agents.

## 1. Introduction

Macroalgae contain large amounts of polysaccharides in their cell walls. A number of these, such as carrageenans, alginates and agar, are widely used by food and cosmetic industries. Furthermore, other metabolites, such as fatty acids, steroids, carotenoids, lectins, mycosporine-like amino acids, halogenated compounds, polyketides, and toxins, as well as other sulfated polysaccharides, make these organisms an economically important product and the focus of considerable biomedical research [1].

Marine macroalgae are usually classified based on their characteristic phytopigment, such as Rhodophyta (red algae), Phaeophyta (brown algae), and Chlorophyta (green algae). Each class also exhibits sulfated polysaccharides. Sulfated galactans are found mainly in Rhodophyta [2,3]. Chlorophyta contains polydisperse heteropolysaccharides, although homopolysaccharides may also be found [4]. Fucan is a term used to denote a polysaccharide family rich in sulfated l-fucose, typical of Phaeophyta. Fucans may be present in the form of homopolymers, called homofucans or fucans, or as heteropolymers, termed fucoidans or, more appropriately, heterofucans [5].

The structure of fucans varies according to algal species. In spite of a large number of studies attempting to determine the fine structure of fucans, only a few examples of regularity were found. The linkages, branching, sulfate positions and composition of monosaccharides differ significantly, and the relationship between structure and biological activity has yet to be established [6]. Therefore, each new sulfated polysaccharide purified from brown seaweed is a new compound with a unique structure and potential novel biological activities [7].

The literature reports a large diversity of biomedical activities associated with fucans, such as antithrombotic, antiviral, antitumoral, immunomodulatory, hypolipidemic, anticomplementary, and antioxidant. They also prevent hepatopathy, renalpathy, and uropathy. Notably, anticoagulant activity is the most widely studied [5].

Urbanization, ageing, and global lifestyle changes have made cardiovascular diseases the leading cause of disability and mortality worldwide. The underlying pathology is atherosclerosis, which develops over many years and is usually advanced by the time it is diagnosed [8]. Modification of risk factors reduces mortality and morbidity, and therapy using anticoagulant agents has been effective in preventing thromboembolic disorders [9].

Unfractioned heparins and their low weight derivatives are the most widely used anticoagulants. However, these compounds have several side effects, such as frequent activated partial thromboplastin time monitoring, variable anticoagulant effects, the inability to inhibit clot-bound thrombin and the occurrence of thrombocytopenia, all of which have led to the search for alternative sources of anticoagulant agents. In this context, sulfated polysaccharides from algae represent a promising treatment option [10].

In recent years, the search for natural antioxidant compounds has also gained considerable attention. Oxidative stress has been linked to a wide range of diseases, age-related degenerative and inflammatory conditions such as diabetes, atherosclerosis, carcinogenesis [11], amyotrophic lateral sclerosis, as well as Alzheimer’s and Parkinson’s diseases [12]. Moreover, synthetic antioxidants such as butylated hydroxyanisole (BHA), butylated hydroxytoluene (BHT), tertiary butyl hydroquinone (TBHQ) and propyl gallate (PG) are used by the food industry to expand the shelf life of their products. These food additives prevent lipid peroxidation, although they have been reported to exert possible toxic and carcinogenic effects on health [13]. In recent years, algal sulfated polysaccharides, especially those extracted from Phaeophyta, have been demonstrated to play an important role as free-radical scavengers and antioxidants in the prevention of oxidative damage in living organisms [2,14].

Recently, we obtained a polysaccharide-rich extract from *Canistrocarpus cervicornis*, whose basionym is *Dictyota cervicornis*. The extract exhibited a range of biological activities, including anticoagulant and antioxidant activities [2]. However, biological activities of the purified sulfated polysaccharides from *C. cervicornis* have not been examined.

Thus, the purpose of the present study was to obtain sulfated polysaccharides from *C. cervicornis* and to evaluate their *in vitro* anticoagulant and antioxidant activities. Our results show several noteworthy differences in the activity of polysaccharides from those *C. cervicornis*. These are likely related to differences in the chemical structure of these compounds. This work focused on selecting the most active sulfated polysaccharide samples for further study as potential novel drugs for thrombosis and/or antioxidant therapy.

## 2. Results and Discussion

### 2.1. Chemical Analyses

We used a low cost and widely reproducible methodology, which combined proteolysis and sequential acetone precipitation, to obtain six acidic polysaccharides from the brown seaweed *C. cervicornis* (denominated CC-0.3, CC-0.5, CC-0.7, CC-1.0, CC-1.2, and CC-2.0). The staining pattern of the polysaccharides on the agarose gel electrophoresis with toluidine blue revealed that all fractions consisted of sulfated polysaccharides (data not shown).

Chemical analysis and sulfated polysaccharide yield are summarized in Table 1. Yield ranged from 10.6% (CC-1.2) to 23.5% (CC-2.0). Low protein contamination in all fractions was caused by the proteolytic enzyme used during polysaccharide extraction. CC-0.3 exhibited the lowest sugar content (12.2%) and CC-1.0 the highest (41.8%). Furthermore, CC-0.7 and CC-2.0 had the highest sulfate content (20.1% and 19.2%, respectively). With respect to total sugar, sulfate and protein contents, the sum of the three components found in the heterofucans does not approach 100%, varying from 15.6% to 58.6%. This is due to the fact that these polymers are highly hygroscopic, absorbing water from the atmosphere very quickly after lyophilization. Furthermore, because of the negative charges of sulfate clusters and glucoronic acids, metals are not eliminated from fucan structures, even after dialysis. Another important point is the conformation exhibited by these polymers in aqueous solutions, which may capture cations within their structures.

When sulfate/total sugar ratio was determined, CC-2.0 and CC-0.5 showed the highest and lowest ratios, respectively.

Sugar composition of polysaccharides (Table 1) was obtained by hydrolysis with 2 M HCl for 2 h, producing the best sugar yields. Data for this hydrolysis condition are shown in Table 1. Data show that glucuronic acid was the major component present in CC-0.3, CC-0.5, CC-0.7, and CC-1.0, whereas galactose was the primary component of CC-1.2 and CC-2.0. Glucose was found only in CC-0.3. Thus, it is clear that the relative amounts of these sugars vary according to the fucan extracted.

Data indicated that the brown seaweed *C. cervicornis* biosynthesizes a complex system of sulfated polysaccharides, six of which (from CC-0.3 to CC-2.0) were obtained after extraction and fractionation. In addition, sulfate and l-fucose were found in all polysaccharides. The manner in which they were obtained, as well as sugar composition, indicated that *C. cervicornis* synthesizes at least six families of sulfated heterofucans: four glucuronofucans (CC-0.3, CC-0.5, CC-0.7 and CC-1.0) and two glucoronogalactofucans (CC-1.2 and CC-2.0).

A number of studies showed that brown algae synthesize more than one type of fucan, such as *Laminaria japonica* [15] and *Sargassum stenophyllum* [16]. Using the methodology described in this paper, we extracted three fucans from *Dictyota mertensiis*, *Padina gymnospora* [17] and *Spatoglossum schroederi* [18] and five fucans from *Dictyota menstrualis* [19], all of which are Dictyotales such as *C. cervicornis*. Thus, the profile encountered was expected, since the presence of heteropolymers is very common in sulfated polysaccharides extracted from brown algae [20].

Infrared spectroscopy has been shown to be a powerful tool for showing similarities between compounds. Table 2 illustrates the main bands observed in the IR spectra of sulfated polysaccharides from *C. cervicornis*.

Characteristic sulfate absorptions were identified in the FT-IR spectra of heterofucans: bands around 1239–1247 cm^−1^ for asymmetric S=O stretching vibration [21] and bands around 1037–1071 cm^−1^ for symmetric C–O vibration associated with a C–O–SO_3_ group [14]. The peaks at 820–850 cm^−1^ were caused by the bending vibration of C–O–S [22].

In addition, all fractions showed signals at 3423–3443 cm^−1^ and around 2920 cm^−1^ from the stretching vibration of O–H and C–H, respectively [23,24]. The bands around 1638–1654 cm^−1^ were caused by the carboxyl group of uronic acid [25].

### 2.2. Anticoagulant Activity

Several articles showed the presence of bioactive sulfated polysaccharides extracted from a number of algae [26]. There are a large number of literature reports about the anticoagulant activity of fucans from brown algae. We recently obtained a polysaccharide-rich extract from *C. cervicornis* that exhibited anticoagulant and antioxidant activities [2]. In this study, we evaluated the sulfated polysaccharide bioactivity of this seaweed, since the use of this rich natural resource seems to be essential to producing an alternative drug in the biomedical industry.

**Figure 1:**
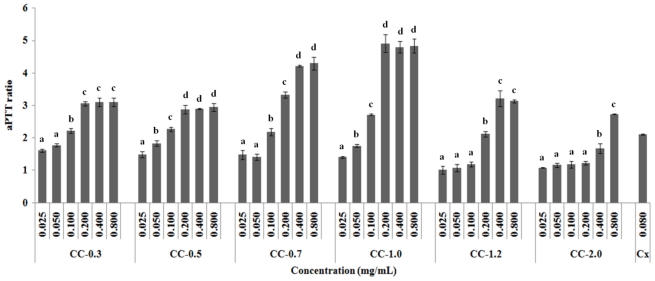
Anticoagulant activity by aPTT test. Results were expressed as aPTT ratio, obtained by dividing clotting time achieved with the anticoagulant by that obtained with the control. Data are expressed as means ± standard deviation of four determinations; ^a,b,c,d^ Different letters indicate a significant difference between each concentration of the same sulfated polysaccharide using one-way Anova followed by the Student-Newman-Keuls test (*p* < 0.05). Cx: Clexane^®^.

The *in vitro* anticoagulant activity of the heterofucans was evaluated by prothrombin time (PT) and activated partial thromboplastin time (aPTT) coagulation assays. No clotting inhibition was observed in the PT test with any of the samples at the concentrations assayed (data not shown). On the other hand, aPTT was prolonged by all polysaccharides in a dose-dependent manner, with respect to the control (Figure 1).

Heterofucans CC-0.3, CC-0.5, CC-0.7, and CC-1.0 doubled aPPT with only 0.1 mg/mL of plasma, a result similar to that of Clexane^®^, a commercial low molecular weight heparin that had the same effect with 0.08 mg/mL of plasma. This high anticoagulant activity was also observed when sulfated polysaccharide-rich extract from *C. cervicornis* was evaluated in an aPTT assay [2]. In addition, heterofucans CC-0.7 and CC-1.0 exhibited the greatest anticoagulant activity, with aPTT ratio of 4.22 and 4.91 with only 0.2 mg/mL and 0.4 mg/mL of plasma, respectively.

*In vitro* anticoagulant activity suggests that these heterofucans do not act by the extrinsic coagulation pathway but rather by inhibiting molecular targets belonging to the intrinsic and/or common coagulation pathways. Previous investigations have shown that the greatest anticoagulant activity of fucans is mediated by this pathway [5].

No correlation was observed between sulfate content and anticoagulant activities in our study. These data are in agreement with those of several other investigations, clearly showing that the anticoagulant effect of fucans is stereospecific and that sulfation site and/or glycosidic linkage affects this activity much more than their charge density or sulfate content [27,28].

### 2.3. Antioxidant Activity

Marine algae inhabit primarily intertidal areas, a harsh environment where they are subjected to repeated immersion and emersion due to tidal fluctuations. As a result, they are exposed twice daily to a wide range of environmental stress, including exposure to ultraviolet radiation, rapid temperature fluctuations, osmotic stress, and desiccation [29,30]. Some of these factors contribute to free radical generation. However, these organisms are not seriously damaged by the presence of these reactive species, indicating that they possess protective mechanisms mediated by enzymes or antioxidant compounds [1,30]. Corroborating these data, several sulfated polysaccharides from marine algae have been recently described as potential antioxidant compounds [2,13–15].

In the present study, *in vitro* antioxidant activity of sulfated polysaccharides from *C. cervicornis* was evaluated using the following assays: total antioxidant capacity, superoxide radical scavenging, hydroxyl radical scavenging and metal-chelating property.

#### 2.3.1. Total Antioxidant Capacity

**Figure 2:**
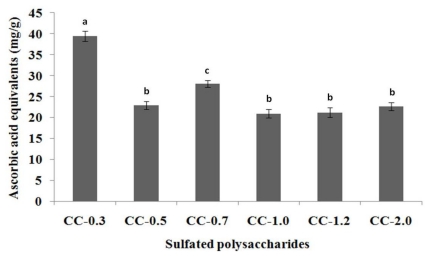
Total antioxidant capacity of sulfated polysaccharides extracted from the marine brown seaweed *C. cervicornis*. The results are expressed as ascorbic acid equivalents. Each value is the mean ± SD of three determinations: ^a,b,c^ Different letters indicate a significant difference (*p* < 0.05) between sulfated polysaccharides.

As total antioxidant capacity is indicative of oxidative stress or increased susceptibility to oxidative damage, this assay was used to determine the antioxidant potential of polysaccharides from *C. cervicornis* (Figure 2).

Total antioxidant capacity of sulfated polysaccharides ranged from 20.9 (CC-1.0) to 39.4 mg/g (CC-0.3) of ascorbic acid equivalents. Acetone fractionation increased total antioxidant capacity of the heterofucans, since sulfated polysaccharide-rich extract from *C. cervicornis* activity was not higher than 20 mg/g of ascorbic acid equivalent [2]. In a previous study using extracts from seaweeds *Padina tetrastomatica* and *Turbinaria conoides*, Chandini and co-workers found 9.79 and 9.65 mg/g of ascorbic acid equivalent, respectively, which was considered an elevated total antioxidant capacity [31]. Thus, the values detected here for sulfated polysaccharides from *C. cervicornis* are extremely interesting, leading us to further testing of different antioxidant assays to determine their possible antioxidant mechanisms.

#### 2.3.2. Hydroxyl and Superoxide Radical Scavenging

Hydroxyl radicals and superoxide anions are reactive oxygen species (ROS) implicated in cell damage. The occurrence of oxidative stress is correlated with numerous pathologies, including neurodegenerative diseases, ischemic or traumatic brain injuries, cancer, diabetes, liver injury, and AIDS [11].

The results of hydroxyl and superoxide scavenging activity are exhibited in Table 3. All sulfated polysaccharides extracted from *C. cervicornis* showed low hydroxyl radical scavenging activity, not exceeding 3% inhibition.

The absence or presence of low activities in the hydroxyl radical scavenging assay is common in sulfated polysaccharides extracted from brown algae [2,5]. This shows that hydroxyl radical scavenging is probably not the major antioxidant mechanism of these heterofucans.

In the superoxide scavenging assay, fucan CC-1.0 showed the lowest antioxidant activity up to the maximum concentration evaluated. CC-0.3, CC-0.5, CC-0.7, and CC-2.0 had only moderate activities, ranging from 13.0% (CC-0.5) to 18.4% (CC-0.7) at 0.1 mg/mL. Heterofucan CC-1.2 deserves special attention, since it had excellent superoxide radical scavenging activity (43.1%) at 0.1 mg/mL, higher than gallic acid, a positive control, at the same concentration (41.8%) and higher than the sulfated polysaccharide-rich extract from *C. cervicornis* [2].

The literature has systematically reported several sulfated polysaccharides extracted from algae with superoxide radical scavenging activity [2,14,32]. The reactivity of the superoxide anion is too low and its biological damage is attributed to indirect effects; for example, from its reaction with hydrogen peroxide, producing hydroxyl radical [33]. On the other hand, the hydroxyl radical has a remarkably short half-life (10^−10^ s), making it the most reactive and harmful ROS. Hydroxyl radical-induced damage can be prevented in two ways: by suppressing hydroxyl radical generation or scavenging the hydroxyl radical generated [32]. Thus, sulfated polysaccharides extracted from *C. cervicornis*, especially in CC-1.2, may prevent damage caused by this potent reactive radical, by inactivating the superoxide radical, one of its precursors.

#### 2.3.3. Chelating Effect on Ferrous Ions

Antioxidants inhibit interaction between metals and lipids through the formation of insoluble metal complexes with ferrous ion or generation of steric resistance. Furthermore, transition metal ions can react with H_2_O_2_, generating hydroxyl radicals, the most reactive free radicals [33].

**Figure 3:**
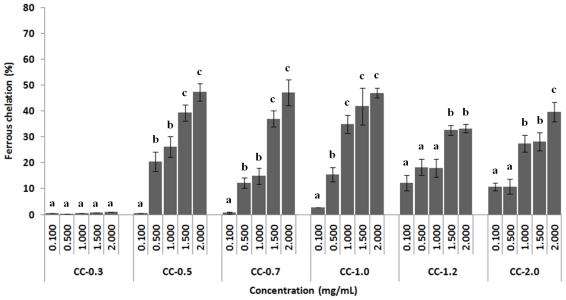
Ferrous chelating activity of sulfated polysaccharides from the brown seaweed *C. cervicornis*. Each value is the mean ± standard deviation of three determinations: ^a,b,c^ Different letters indicate a significant difference (*p* < 0.05) between each concentration of the same sulfated polysaccharide.

The chelating effect on ferrous ions exhibited by sulfated polysaccharides extracted from *C. cervicornis* is shown in Figure 3. The results revealed that heterofucan CC-0.3 did not display significant ferrous chelating capacity, while CC-1.2 and CC-2.0 showed moderate activity at the maximum concentration tested, chelating 33.3% and 39.7% of ferrous ions, respectively. CC-0.5, CC-0.7, and CC-1.0 showed similar dose-dependent activity, reaching maximum activity (about 47.0%) at a concentration of 2.0 mg/mL. This was only 1.8 times lower than EDTA activity at the same concentration under the same experimental conditions (data not shown). The purification process did not increase the chelating effect of the heterofucans compared to the chelating effect of sulfated polysaccharide-rich extract from *C. cervicornis* [2].

The results obtained by heterofucans, with the exception of CC-0.3, were higher than those shown previously by Costa and co-workers where a sulfated polysaccharide-rich extract from *C. cervicornis* had a chelating capacity of about 30.0% at a concentration of 2.0 mg/mL [2]. These heterofucans still had superior activity to that of sulfated polysaccharides extracted from *Laminaria japonica* [34], low molecular and high sulfated derivatives from polysaccharides extracted from *Ulva pertusa* [32], and sulfated polysaccharide-rich extracts from the algae *Dictyopteris delicatula*, *Dictyota menstrualis*, *Sargassum filipendula*, *Spatoglossum schroederi*, *Gracilaria caudata*, *Caulerpa cupressoides* and *Codium isthmocladum* [2].

Sulfate content seems to influence the antioxidant activity of sulfated polysaccharides extracted from algae [13,32]; however, our data did not show a correlation between sulfate content and antioxidant activity, once again indicating that sulfation site is much more important than sulfate content.

The recent abundant evidence suggests the involvement of reactive oxygen species (ROS) in the signaling of various cellular events. Attack by ROS has been implicated in the pathogenesis of a number of disorders and diseases such as diabetes, atherosclerosis, and other vascular ailments, and the use of various antioxidants is known to inhibit these events [35]. In addition, anticoagulant drugs are also used in the treatment of these same diseases. In this study, we found several anticoagulant and antioxidant heterofucans from *C. cervicornis* that will be selected for further studies as potential novel multipotent anticoagulant/antioxidant drugs.

## 3. Experimental Section

### 3.1. Materials

Iron(II) sulfate, potassium ferricianyde, sulfuric acid and acetonitrile were obtained from Merck (Darmstadt, Germany). Nitro Blue Tetrazolium (NBT), monosaccharides, methionine and ammonium molybdate were purchased from Sigma-Aldrich Co. (St. Louis, USA). All other solvents and chemicals were of analytical grade.

### 3.2. Extraction of Sulfated Polysaccharide Fractions

The Phaeophyta *Canistrocarpus cervicornis* was collected at Búzios Beach, Nísia Floresta, Brazil. Immediately after collection, the alga was identified by Dr. Eliane Marinho from the Centro de Biociências at Universidade Federal do Rio Grande do Norte (UFRN), in Natal, Brazil. The alga was stored in our laboratory and dried at 50 °C under ventilation in an oven, ground in a blender and incubated with acetone to eliminate lipids and pigments. About 90 g of powdered alga was suspended with five volumes of 0.25 M NaCl and the pH was adjusted to 8.0 with NaOH. Next, 900 mg of Prolav 750 (Prozyn Biosolutions, São Paulo, SP, Brazil), a mixture of alkaline proteases, was added for proteolytic digestion. After incubation for 24 h at 60 °C under agitation and periodical pH adjustments, the mixture was filtered through cheesecloth. The filtrate was fractionated by precipitation with acetone as follows: 0.3 volumes of ice-cold acetone was added to the solution under gentle agitation and maintained at 4 °C for 24 h. The precipitate formed was collected by centrifugation (10,000 × g, 20 min), vacuum dried, resuspended in distilled water, and analyzed. The operation was repeated by adding 0.5, 0.7, 1.2, 1.5, and 2.0 volumes of acetone to the supernatant.

### 3.3. Agarose Gel Electrophoresis

Agarose gel electrophoresis of the acidic polysaccharides was performed in 0.6% agarose gel (7.5 cm × 10 cm × 0.2 cm thick) prepared in 0.05 M 1.3-diaminopropane acetate buffer pH 9.0, as previously described [7]. Aliquots of the polysaccharides (about 50 μg) were applied to the gel and subjected to electrophoresis. The gel was fixed with 0.1% cetyltrimethylammonium bromide solution for 2 h, dried, and stained for 15 min with 0.1% toluidine blue in 1% acetic acid in 50% ethanol. The gel was then destained with the same solution without the dye.

### 3.4. Chemical Analysis and Monosaccharide Composition

Total sugars were estimated by the phenol-H_2_SO_4_ reaction [36] using l-fucose as standard. Sulfate content was determined according to the gelatin-barium method [37], using sodium sulfate (1 mg/mL) as standard and after acid hydrolysis of the polysaccharides (4 M HCl, 100 °C, 6 h). Protein content was measured using Spector’s method [38].

The polysaccharides were hydrolyzed with 0.5, 1, 2, and 4 M, respectively, for various lengths of time (0.5, 1, 2 and 4 h) at 100 °C. Reducing sugars were determined using the Somogyi-Nelson method [39]. After acid hydrolysis, sugar composition was determined by a LaChrom Elite^®^ HPLC system from VWR-Hitachi with a refractive index detector (RI detector model L-2490). A LichroCART^®^ 250-4 column (250 mm × 40 mm) packed with Lichrospher^®^ 100 NH_2_ (5 μm) was coupled to the system. The sample mass used was 0.2 mg and analysis time was 25 min. The following sugars were analyzed as references: arabinose, fructose, fucose, galactose, glucose, glucosamine, glucuronic acid, mannose, and xylose.

### 3.5. Fourier Transformed Infrared Spectroscopy (FT-IR)

Sulfated polysaccharides (5 mg) were mixed thoroughly with dry potassium bromide. A pellet was prepared and the infrared spectra between 500 and 4000 cm^−1^ was measured on a Thermo-Nicolet Nexus 470 ESP FT-IR spectrometer. Thirty-two scans at a resolution of 4 cm^−1^ were averaged and referenced against air.

### 3.6. Anticoagulant Activity

Prothrombin time (PT) and activated partial thromboplastin time (aPTT) coagulation assays were performed with a coagulometer as described earlier [19] and measured using normal citrate-treated human plasma. All assays were performed in duplicate and repeated at least three times on different days (*n* = 6). The results were expressed as aPTT ratio, which was determined as follows: aPTT control time/aPTT sample time.

### 3.7. Antioxidant Activity

Four assays were performed to analyze the antioxidant activity of the sulfated polysaccharides obtained: total antioxidant capacity, hydroxyl radical scavenging, superoxide radical scavenging, and ferric chelating, as previously described [2].

#### 3.7.1. Determination of Total Antioxidant Capacity

This assay is based on the reduction of Mo(VI) to Mo(V) by sulfated polysaccharides and subsequent formation of a green phosphate/Mo(V) complex at acidic pH. Tubes containing sulfated polysaccharides and reagent solution (0.6 M sulfuric acid, 28 mM sodium phosphate and 4 mM ammonium molybdate) were incubated at 95 °C for 90 min. After the mixture had cooled to room temperature, the absorbance of each solution was measured at 695 nm against a blank. Total antioxidant capacity was expressed as ascorbic acid equivalent.

#### 3.7.2. Hydroxyl Radical Scavenging Activity Assay

The scavenging activity of seaweed polysaccharides against the hydroxyl radical was investigated using Fenton’s reaction (Fe^2+^ + H_2_O_2_ → Fe^3+^ + OH^−^ + OH^•^). These results were expressed as inhibition rate. Hydroxyl radicals were generated using 3 mL sodium phosphate buffer (150 mM, pH 7.4), which contained 10 mM FeSO_4_·7H_2_O, 10 mM EDTA, 2 mM sodium salicylate, 30% H_2_O_2_ (200 mL) and varying polysaccharide concentrations. In the control, sodium phosphate buffer replaced H_2_O_2_. The solutions were incubated at 37 °C for 1 h, and the presence of the hydroxyl radical was detected by monitoring absorbance at 510 nm. Gallic acid was used as positive control.

#### 3.7.3. Superoxide Radical Scavenging Activity Assay

This assay was based on the capacity of sulfated polysaccharides to inhibit the photochemical reduction of nitroblue tetrazolium (NBT) in the riboflavin-light-NBT system. Each 3 mL of reaction mixture contained 50 mM phosphate buffer (pH 7.8), 13 mM methionine, 2 mM riboflavin, 100 mM EDTA, NBT (75 mM) and 1 mL sample solution. After the production of blue formazan, the increase in absorbance at 560 nm after 10 min illumination from a fluorescent lamp was determined. The entire reaction assembly was enclosed in a box lined with aluminum foil. Identical tubes with the reaction mixture were kept in the dark and served as blanks. Gallic acid was used as positive control.

#### 3.7.4. Ferric Chelating

The ferrous ion chelating ability of samples was investigated using the following methodology: sulfated polysaccharides at different concentrations were applied with the reaction mixture, which contained FeCl_2_ (0.05 mL, 2 mM) and ferrozine (0.2 mL, 5 mM). The mixture was shaken and incubated for 10 min at room temperature and absorbance of the mixture was measured at 562 nm against a blank. EDTA was used as positive control.

### 3.8. Statistical Analysis

All data were expressed as mean ± standard deviation. Statistical analysis was done by one-way Anova using the SIGMAStat 2.01 software. Student-Newmans-Keuls post-tests were performed for multiple group comparison. In all cases statistical significance was set at *p* < 0.05.

## 4. Conclusions

We obtained six types of anticoagulant and antioxidant heterofucans from the brown alga *C. cervicornis*. These were denominated CC-0.3, CC-0.5, CC-0.7, CC-1.0, CC-1.2, and CC-2.0. Heterofucans CC-0.3, CC-0.5, CC-0.7 and CC-1.0 were able to double aPPT with only 0.1 mg/mL of plasma, a result similar to that obtained with Clexane^®^. Indeed, CC-0.7 and CC-1.0 had the greatest anticoagulant activity. All the heterofucans exhibited appreciable total antioxidant capacity, low hydroxyl radical scavenging activity, good superoxide radical scavenging efficiency (except CC-1.0) and excellent ferrous chelating ability (except CC-0.3). With such strong concomitant antioxidant and anticoagulant activities, several heterofucans, mainly CC-0.7 and CC-1.2, were identified as potential multipotent drugs.

## Figures and Tables

**Table 1:** Chemical composition of polysaccharides extracted from the brown seaweed *Canistrocarpus cervicornis*. S/TSU ratio: Sulfate/Total sugar ratio; Fuc: fucose; Gluc acid: glucuronic acid; Gal: galactose; Xyl: xylose; Man: mannose; Gluc: glucose; -: Traces; n.d.: not detected.

Sulfated Polysaccharides	Yield a (%)	Total sugar (%)	Sulfate (%)	S/TSU ratio (%/%)	Protein (%)	Molar Ratio					
						Fuc	Gluc Acid	Gal	Xyl	Man	Gluc
**CC-0.3**	14.8	12.2	2.8	0.2	0.6	1.0	2.0	0.5	0.5	0.5	0.5
**CC-0.5**	12.1	33.4	3.9	0.1	0.5	1.0	2.0	-	0.5	-	-
**CC-0.7**	21.3	33.2	19.2	0.6	0.4	1.0	1.5	-	0.5	-	n.d.
**CC-1.0**	17.7	41.8	16.5	0.4	0.3	1.0	1.0	0.5	0.5	-	n.d.
**CC-1.2**	10.6	21.8	7.8	0.4	0.2	1.0	1.0	2.5	0.5	-	n.d.
**CC-2.0**	23.5	19.9	20.1	1.0	0.2	1.0	1.0	2.0	0.5	0.5	n.d.

a All polysaccharides obtained by acetone precipitation were dried and weighed and total polysaccharides corresponded to 100%.

**Table 2:** IR spectrum data of six heterofucans from the brown seaweed *Canistrocarpus cervicornis*.

Sulfated Polysaccharides	IR (KBr) (cm^−1^)
**CC-0.3**	3441, 1646, 1247, 1037, 820
**CC-0.5**	3428, 1650, 1233, 1071, 850
**CC-0.7**	3434, 1642, 1247, 1064, 833
**CC-1.0**	3423, 1644, 1231, 1065, 841
**CC-1.2**	3431, 1641, 1239, 1042, 827
**CC-2.0**	3443, 1649, 1251, 1056, 839

**Table 3:** Hydroxyl and superoxide scavenging activity of sulfated polysaccharides from the brown seaweed *Canistrocarpus cervicornis*. Each value is the mean ± SD of three determinations: ^a,b,c,d^ Different letters indicate a significant difference (*p* < 0.05) between each concentration of the same sulfated polysaccharide.

Sulfated polysaccharides	Concentration (mg/mL)	Scavenging (%)	
		OH^•^	O_2_^−^
**CC-0.3**	0.010	0.0 ± 0.0 ^a^	3.9 ± 1.4 ^a^
	0.025	0.0 ± 0.0 ^a^	18.1 ± 1.3 ^b^
	0.050	0.9 ± 0.1 ^b^	18.0 ± 0.9 ^b^
	0.100	1.0 ± 0.2 ^b^	18.0 ± 1.8 ^b^
**CC-0.5**	0.010	0.0 ± 0.0 ^a^	3.4 ± 0.6 ^a^
	0.025	0.0 ± 0.0 ^a^	8.3 ± 1.2 ^b^
	0.050	0.1 ± 0.1 ^a^	13.3 ± 0.9 ^c^
	0.100	0.5 ± 0.1 ^b^	13.0 ± 1.8 ^c^
**CC-0.7**	0.010	0.0 ± 0.0 ^a^	3.1 ± 1.3 ^a^
	0.025	0.0 ± 0.0 ^a^	18.1 ± 1.3 ^b^
	0.050	0.9 ± 0.2 ^b^	18.2 ± 6.1 ^b^
	0.100	0.9 ± 0.0 ^b^	18.4 ± 1.2 ^b^
**CC-1.0**	0.010	0.0 ± 0.0 ^a^	0.8 ± 0.5 ^a^
	0.025	0.0 ± 0.0 ^a^	0.7 ± 1.4 ^a^
	0.050	2.9 ± 0.2 ^b^	0.8 ± 1.7 ^a^
	0.100	3.0 ± 0.1 ^b^	0.3 ± 2.1 ^a^
**CC-1.2**	0.010	0.0 ± 0.0 ^a^	9.2 ± 1.0 ^a^
	0.025	0.0 ± 0.0 ^a^	14.1 ± 1.9 ^b^
	0.050	2.9 ± 0.1 ^b^	24.2 ± 3.7 ^c^
	0.100	3.0 ± 0.1 ^b^	43.1 ± 2.1 ^d^
**CC-2.0**	0.010	0.0 ± 0.0 ^a^	3.4 ± 0.7 ^a^
	0.025	0.0 ± 0.0 ^a^	6.0 ± 1.4 ^b^
	0.050	2.4 ± 0.2 ^b^	13.4 ± 0.8 ^c^
	0.100	2.9 ± 0.1 ^b^	13.1 ± 1.7 ^c^
**Gallic acid**	0.100	43.6 ± 2.4	41.8 ± 4.7
